# Rationales, design and recruitment for the Elfe longitudinal study

**DOI:** 10.1186/1471-2431-9-58

**Published:** 2009-09-21

**Authors:** Stéphanie Vandentorren, Corinne Bois, Claudine Pirus, Hélène Sarter, Georges Salines, Henri Leridon, the Elfe team

**Affiliations:** 1 Département santé environnement, Institut de veille sanitaire, Saint Maurice, France; 2 Institut National des études démographiques, Paris, France; 3 U822 'Epidémiologie, Démographie et Sciences Sociales', INSERM, Le Kremlin-Bicêtre, France

## Abstract

**Background:**

Many factors act simultaneously in childhood to influence health status, life chances and well being, including pre-birth influences, the environmental pollutants of early life, health status but also the social influences of family and school. A cohort study is needed to disentangle these influences and explore attribution.

**Methods:**

Elfe will be a nationally representative cohort of 20 000 children followed from birth to adulthood using a multidisciplinary approach. The cohort will be based on the INSEE Permanent Demographic Panel (EDP) established using census data and civil records. The sample size has been defined in order to match the representativeness criteria and to obtain some prevalence estimation, but also to address the research area of low exposure/rare effects. The cohort will be based on repeated surveys by face to face or phone interview (at birth and each year) as well as medical interview (at 2 years) and examination (at 6 years). Furthermore, biological samples will be taken at birth to evaluate the foetal exposition to toxic substances, environmental sensors will be placed in the child's homes. Pilot studies have been initiated in 2007 (500 children) with an overall acceptance rate of 55% and are currently under progress, the 2-year survey being carried out in October this year.

**Discussion:**

The longitudinal study will provide a unique source of data to analyse the development of children in their environment, to study the various factors interacting throughout the life course up to adulthood and to determine the impact of childhood experience on the individual's physical, psychological, social and professional development.

## Background

Many factors act simultaneously in childhood to influence health status, life chances and well being, including pre-birth influences, the environmental pollutants of early life, health status but also the social influences of family and school.

Nevertheless, scientific knowledge concerning the critical ages when health effects are triggered or concerning the etiological fraction of several risk factors in the morbidity and the mortality of children remain incomplete. Increased efforts in research and surveillance are necessary if we want to have more efficient prevention programs.

The protocol of the "Etude Longitudinale Française depuis l'Enfance" (ELFE) (French longitudinal study of children) was the final result of several projects, promoted by different research and government institutions. In 2005 these projects converged towards a unique multidisciplinary national study called the "Etude Longitudinale Française depuis l'Enfance" (ELFE) (French longitudinal study of children).

Before building this final protocol, and because of the inherent complexity in its elaboration, this study underwent many planning and development stages before reaching its current objectives and design.

In the late 1990s, many public institutions and government authorities, including the National Institute of Demographic Studies (INED, Institut national d'études démographiques), the Statistical Office (INSEE), the Ministry of Health (Ministère de la santé), the Ministry of Social Affairs (Ministère des affaires sociales) and the Council for Employment, Income and Social Cohesion (Conseil de l'emploi, des revenus et de la cohésion sociale) expressed their concerns about the lack of French longitudinal studies, notably on child development. In 2002, the idea arose of setting up an extensive cohort of 20,000 children in France, to be followed from birth to adulthood.

In parallel, in June 2004, France adopted a national health and environment action plan (Plan national santé environment, PNSE) which addressed questions raised by the French civil society regarding the short- and medium-term health impacts of exposure to environmental pollutants. Among its priorities was the creation of a large-scale epidemiological cohort of children, contributing to both research and surveillance in the field of environmental health. The main objectives of this project were twofold: first, to measure exposures to pollutants (chemical, biological agents, and physical factors), and contamination levels in biological samples during pregnancy and early childhood; second, to describe the health status of children and analyze the links between health and pollutant exposures at critical periods in child development and identify which periods are the most critical. The design and implementation of this project was entrusted to the French Institute for Public Health Surveillance (Institut de Veille Sanitaire, InVS).

In June 2005, the institutions involved in these projects decided to merge their efforts into a single national project entitled Etude Longitudinale Française depuis l'Enfance (ELFE) (French longitudinal study of childhood). It is currently managed by a consortium (Groupe d'interêt scientifique, GIS) which liaises with the main project partners: INED, InVS, INSERM, INSEE, and government bodies such as DGS, DREES (Ministry of health), DEPP (Ministry of education) and CNAF (National Family Allowance Office). The GIS includes a strategic steering committee in charge of project governance composed of representatives of GIS institutions, a scientific council including national and intenational scientists and a project team composed of coordinators, scientific, technical and administrative staff as well as scientists from other institutions.

National and International partnerships have been developed through collaboration with existing national cohorts (EDEN [1], PELAGIE [2], TIMOUN), foreign cohorts (INMA [3], MoBa [4] Millenium Cohort Study [5]...) and networks (EUCCONET, I4C [6], Enrieco).

Two pilot studies were carried out to validate the data collection methods to be used for the first year of the study when it commences. The first took place in April 2007 in Bourgogne and Picardie regions and the second in October 2007 in Seine Saint Denis county and Rhône Alpes region, except Rhône department. These studies provided an opportunity to estimate not only the study's participation acceptance rate but also to assess its field feasibility. Moreover the second pilot study has been useful to test procedures of collection and bio-banking of biological samples that are scheduled at the end of 2010.

The official starting date for enrolments in the cohort is planned for the beginning of 2011.

## Methods

### Study objectives

The purpose of the ELFE project is to build a nationally representative cohort of 20,000 children to be followed from birth to adulthood using a multidisciplinary approach in order to characterise the relationship between environmental exposures and the socio-economic context on health and behaviours more thoroughly.

The rationale of this cohort arises from the consideration that from conception to teenage years, children pass through various stages of development where their environment whether characterised at the social, economic or familial level, by exposure to environmental pollutants, by nutritional aspects not only play individual roles in determining their future health and behaviours but also interact to produce certain unforeseen health events. To date, these various dimensions have been given only scant attention in epidemiologic research and most available studies in the field simply focus on a single subset of these factors.

The project objectives cover the fields of epidemiology, public health and social sciences.

Among specific objectives, the project will assess the consequences of contamination during pregnancy and early childhood by well-known pollutants (lead, mercury, PCB, pesticides) as well as emerging pollutants (phthalate, etc.) on a child's neuro-cognitive and reproductive development. The study will also explore the adverse consequences of exposure to outdoor air pollution on pregnancy outcomes and on child growth and respiratory diseases.

In the field of social sciences, for example, the study will address the consequences of the family situation (blended families, etc.), life events (divorces and second marriages, etc.), as well as the effects of poverty and social policies on families and interactions with the educational system.

In the field of health, the Elfe project will give a central place to the growth, by examining all the relationships between growth, social, environment's exposures and health events. The results from the ELFE cohort will contribute to update data presented by the WHO child growth standards published in 2006 [7,8]

It will also focus on the development of children in their environment, to study the various factors interacting throughout one's lifetime (family structure, social and physical environment, schooling, health, diet and eating behaviour, etc.) to determine the impact of childhood experience on individuals' social and occupational trajectories.

### The major hypotheses

Although all the research areas can not be foreseen in a study planned for a long follow-up, in the planning phase several major domains have been delineated.

#### 1. Environmental exposure and health

Increased susceptibility to a particular chemical substance may occur in children and depend on the specific physical, toxicological and age-related pharmacokinetic characteristics of the substance as well as the stage of child development [9-11].

The study will thus investigate the effects of environmental conditions and exposures on child health using various innovative approaches. Models will allow us to characterize child exposure to emerging pollutants (phthalates, biocides, etc.) and to evaluate the risk factors using mathematical models, adjusted and re-weighted with respect to the France's population of children. Emphasis will be put on the combined interactions and effects of several neuro-toxic products (lead, mercury, PCB, pesticides) and various endocrinal disturbers. We will develop new biomarkers for exposure, effects and sensitivity in order to determine the associated neurological risks and endocrinal disturbances. The possible implication of various chemicals (lead, mercury, PCBs: polychlorinated biphenyls, pesticides) in endocrine dysfunctions and neurobehavioral changes will be studied, taking into account the result of socioeconomic and lifestyle choices. The impact of long-term exposure to low levels of lead will also be investigated to verify that there no minimum threshold cumulative effect exists which could be responsible for the possible impairment in children's intellectual abilities. Both indoor and outdoor air quality appear to be related to asthma and chronic childhood respiratory diseases [12]. The ELFE study will provide an opportunity to study relationships between outdoor air pollutants (ozone, NO_2_, SO_2_, PM_10_...) and children's respiratory health status.

Exposure to radiation from all sources including UV, radon and medical radiation is a hazard to children's health [13-15]. Individual cumulative exposure to UV rays will be also evaluated in the cohort, in order to study childhood sunburn and the ophthalmologic effects of UV exposure, as well as precancerous lesions (naevi) and melanoma. The health effects (especially leukaemia in childhood) of therapeutic and diagnostic radiation exposure will be explored. For this purpose, the data will be pooled with those of other cohorts through the I4C project [6].

#### 2. Social and nutritional aspects of child's feeding and its relation with development and health

The study will investigate the question of how eating habits are acquired and passed on, along with the link between food, health and child and teenage weight status. Special attention will be given to the analysis of eating behaviour and its effects on development and health. Impact of pre- and post-natal nutrition on obesity and overweight in childhood will be studied, taking into account various other factors such as socio-economic status or physical activity.

The study will also focus on physical growth (obesity), puberty, bone mineralization and insulin resistance syndrome. The cohort will allow us to study the long term consequences of overweight on health status (in particular: respiratory, metabolic, cardiovascular diseases or orthopaedic and fertility disorders) as well as social and school trajectories.

#### 3. Development of updated growth reference curves

The current child growth standards of the French paediatric population are obsolete as it still relies on the reference values based on the paediatric population born in the fifties. Anthropometric measures obtained in a representative subsample of children born between 2010-2011 of the ELFE cohort will allow us to obtain updated and reliable child growth standards. Such values will be compared to the WHO child growth standards published in 2006 that report international growth reference curves of children living in optimal conditions.

#### 4 The child's cognitive development

The emphasis will be placed on gross motor, fine motor, cognitive development and language. The objective will be to explore child's development in relation with his/her family, social, economic environment but also exposure to environmental pollutants. The effect of nutrition, especially early nutrition (between 0 and 2 years old) and the impact of health-related characteristics (such as perinatal characteristics) on child development will also be assessed. Emphasis will be put on the relation between early cognitive and language development and school abilities.

#### 5. Asthma and allergies: incidence, early determinants

Another research topic concerns asthma and allergies, which have become increasingly frequent in industrialized countries over recent years [16]. In France, with a cumulative prevalence of more than 10% in children, asthma remains a major public health problem [17]. We will collect data to estimate the incidence and prevalence of asthma and its different degrees of severity at different ages during childhood and according to socio-economic group. Early determinants of asthma and allergies in childhood will be evaluated, such as environmental factors, socio-economic factors, lifestyle and nutrition. The "hygiene hypothesis" [18] whereby early exposure not only to allergens but also to infectious diseases, immunization and other environmental stimuli appears to reduce the risk of asthma and atopic diseases will also be tested in the ELFE study.

#### 6. Family environment and events and social outcomes

##### Children's Living Conditions

A large number of studies carried out in the United States, Canada, the United Kingdom and in countries in northern Europe have highlighted the importance of living conditions and care of young children on their state of health, cognitive development and scholastic results [19-21]. Beyond the aspects linked to household income, we will study to what degree the familial situation also plays a role: the number of children living with a single parent, situations of multiple residence (which has seen a dramatic increase in recent years), and also how child care duties are divided between the parents [22].

##### Generational relations and first experiences

Confirming results from other studies [23], the longitudinal study SHARE (Survey of Health, Ageing and Retirement in Europe) has recently pointed out that one third of grandparents help out in the care of their grandchildren. The ELFE study will evaluate this assistance and will measure its effect in the context of the transformation of the family structure (see above) as well as geographic distance from relatives [24]. We shall also test the theory of attachment [25], which constitutes the principal theoretical framework used to understand/explore the importance of first relations in the development of a child. According to longitudinal studies carried out in the United States and in Europe [26], individual characteristics and a child's physical and social environments play an important role in the construction of these first relations.

##### The construction of children's sexual identities

Sexual identities are initially developed according to parental behaviour. All studies have shown that the gender division between household and parental work is only slowly changing [27]. We shall investigate activities connected with health, hygiene and the body in reference to work of anthropologists and psychologists [28]. The immediate surroundings of the child, in the form of toys or children's literature which the child may be exposed to, may also play a role in gender identity [29]. Finally, the role of child professionals [30] will also be taken into account.

### Study design

**Figure 1:**
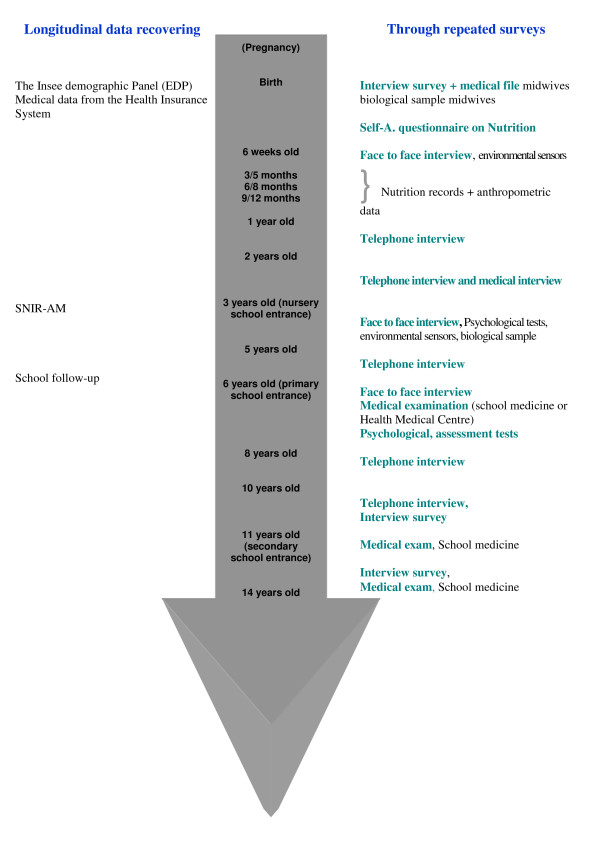
**ELFE Follow-up**.

The design of the cohort is based on an initial enrolment interview of mothers at the child's birth in order to obtain retrospective data about exposures during pregnancy then a prospective follow-up of the child (figure 1). The follow-up of the children is based on data retrieval and record linkage from existing databases (INSEE demographic data, school follow-up, Health Insurance records, National environment database etc.) as well as on several waves of cross-sectional surveys to be carried out at different ages: 6 weeks, 1, 2, 3, 5, 6, 8, 10, 11, 14 years old. These surveys may include biological sampling and will be based on face-to-face interview surveys or telephone interviews, clinical examinations and psychomotor development tests, self-administered questionnaires, measures of the environment, etc.

With respect to active data collection, the first survey, considered as the baseline survey, will take place at maternity hospitals to obtain information about pregnancy, the perinatal period and the mother's and newborn's health at delivery. This early data collection includes a face-to-face interview with the associated midwife and the collection of medical data using the newborn's and mother's medical files as well as a self-administered questionnaire for the mother.

Biological samples will be collected at the maternity hospital to supply information on biomarkers of exposure to pollutants, various nutrients and genetic traits. This survey will be crucial with regard to the final recruitment of children since it is during the stay at the maternity hospital that the parents will decide whether or not to remain in the study. The names and addresses of the children will be collected in order to interview the mother (or a legal guardian) a few weeks later.

The second survey will be conducted by the National Institute for Statistics and Economic Studies (INSEE) and will take place at the infants' homes, 6-8 weeks after birth. It will comprise a one-hour face-to-face interview with the mother (or the legal guardian) and will be completed by a 20-minute telephone interview with the father. Environmental sensors will be placed in some of the infants' homes.

The following repeated surveys will be based on phone interviews. The first of these, at age 1, will focus on health and social topics and the second, at age 2, will detail environmental factors. A face-to-face interview will be conducted at their home when the children are 3 years old and will focus on their development using psychomotor tests. Environmental sensors will also be placed in some children's homes to measure exposure to indoor pollutants. Anthropometrics measures will be taken by health professionnal specially turned out, on a undersample of 2000 children, each year since age one. At age 6, the children will undergo a medical examination and psychomotor tests. Further follow-ups after age 6 have not yet been established.

### Study recruitment

The cohort will be based on the INSEE Permanent Demographic Sample (échantillon démographique permanent, EDP) established using census data and civil records ("Etat Civil" which lists all births, marriages and deaths). Since 1968, all French citizens born on specific days of the year (until now, 4 days in October and, in the future, 16 days throughout the year) have been included in this sample. The EDP is considered a representative sample of the population of metropolitan France with a sampling rate equivalent to 1/100 (4/365) of the population residing in metropolitan France. About 900 000 individual life histories are currently being tracked, offering significant opportunities for social and demographic studies [31].

Our base panel will thus include all children born in hospital maternity units on a total of 16 days, 4 days in each of the 4 different quarters of the inclusion year. This will enable us to constitute a representative sample of births in a particular year so that these children can be easily identified in other data sources.

For practical reasons, the base panel will be restricted to single or twin births. Mothers of children in this base panel will be invited to sign a consent form presenting the general aim of the study and the purpose of the biological samples. Consent for biological sampling and consent for the remaining of the data collection will be dissociated. The participants will be able to withdraw at any time.

An information campaign will be conducted in all maternity units before the beginning of the study. A postal invitation to participate will be sent to all mothers during the 7^th ^month of pregnancy via the newsletter sent by CNAF. Fathers will also be invited to participate. The protocol of the study has been approved by four different ethical approvals for the first steps of the pilots surveys: one from the CNIL (that is the board enforcinglaws on data protection); one from the CNIS (that is the National Council on Statistics Information); one from the CPP (that is the Committee of Persons Protection) and one from the CCTIRS (that is a government comitee consulted on issues on information concerning health research).

The participation acceptance rates in the pilot surveys were respectively 57% (n = 191 deliveries enrolled) in April 2007 (participation of 32/35 maternity units) and 54% (n = 300) in October 2007 (participation of 30/40 maternity units). We expect to improve these rates through better organization and information in the maternity units. Biological sample collection was tested in October 2007 and showed an excellent acceptance rate (90%). Finally biological samples were collected for 80% of the population enrolled in the pilot study (table 1).

**Table 1:** Distribution of main characteristics of deliveries of infants enrolled in the two pilot studies

	**First pilot study** **(338 live deliveries)**		**Second pilot study** **(552 live deliveries)**	
	**n**	**%**	**n**	**%**
Participation rate at maternity	191	57	300	54
Biological samples collected*	-	-	248	82
Parity				
0	58	36	113	37
1	63	39	111	36
2	41	25	82	27
Maternal age				
<20	2	1	4	1
20-24	30	17	34	11
25-29	65	36	89	30
30-34	55	30	118	40
35+	29	16	53	18
Preterm birth	15	8	24	8
Caesarean delivery				
Yes	35	19	53	18
No	147	81	247	82
Birth weight (g) Mean (SD)	3346 (471)		3335 (496)	

* only tested for the second pilot study

### Sample size and anticipated attrition rate

The sample plan and size have been defined to fulfil representativeness criteria and to obtain consistent prevalence estimates of events, but also to increase the probability of observing low exposures or rare effects. As an example, 19100 subjects are required to investigate the prevalence of high lead-poisoning (p = 0.5%) and 13200 subjects are needed to correlate low-antioxidant diet with prematurity risk - with 5% of premature birth and a 1.5% difference between antioxidant extreme quartiles (>p75 vs <p25). The sample size also arise from feasibility issues as the cohort is based on the permanent demographical sampling of the INSEE (EDP). Since the EDP should concern around 36000 children in 2010, it is reasonable to presume that it will be possible to follow at least 20 000 of the children included in EDP (i.e. 55%). Attrition rates will be estimated only for the follow-up of children and mothers across the different waves of surveys as data available from EDP are seldom affected by attrition. However, attrition rates are difficult to forecast because they largely depend on the characteristics of the survey (data collection mode, data retention and tracking plan, subject of the study etc.) and no study exists with the same scope and design as ELFE.

The two pilot studies have already provided us with some results on participation, two months and one year after enrolment in the study respectively. The response rate to the individual parent questionnaire at two months after birth was 86% for mothers and 79% fathers. Major reasons for non response of non-responses were refusals (55%), 31% were impossibility to contact the women, 12% were due to other reasons, and 6% were for unknown reasons. After the two months interview, 7% of the sample expressed a final refusal to continue participation. First results for participation at one year are also available: amongst the parents still in the cohort at this point 82% of mothers (corresponding to 77% of mothers contacted at 2 months) and 70% of fathers (corresponding to 65% of fathers contacted at 2 months) responded to the questionnaire.

Attrition rates may also depend on unwillingness to answer the questionnaire or interview. Non-response can lead to selection bias, and if added to the attrition process [32] can contribute to increasing missing data over time. Consequently, procedures limiting non-response have been planned, including prevention tools (communication based, tracking, incentives etc.) and methods of dealing with non-response through statistical analysis (e.g. reweighting methods [33]).

### Sample collection

#### 1. Environmental and social exposures

##### Demographic and socio-economic data

Most of these data will be available thanks to data retrieval from national databases such as INSEE demographic records (EDP), health insurance data and school records.

The different surveys (including data from interviews with parents or children) will provide additional socio-demographic and contextual information on family, socialization, education and income in order to better identify and quantify the exposure to particular socio-economic conditions (i.e. degree of poverty) and life events (i.e. divorces, change in economic conditions, school insertion etc.)

##### Major environmental exposures

Biological samples will include cord-blood, breast milk, mother's urine and hair and mother's venous blood. Planned measurements concern environmental pollutants in cord blood (lead, selenium), milk (POPs), hair (mercury) and in mother's urine (pesticides, phthalates). Biological samples will be appropriately fractioned and stored so that further analyses will be possible during follow-up.

To evaluate exposure to certain pollutants, environmental sensors will be placed in the children's homes. Furthermore, it will be possible to use data from environmental databases thanks to geo-coding of the addresses. The mother's answers to questions on various environmental exposures included in the different questionnaires will complement these quantitative data.

##### Dietary data

A dietary questionnaire will be filled up by the mothers during the stay in the maternity wards. It is designed to assess food intake under the nutritional, sensorial and social perspective. In addition, stored blood and milk samples will be available for the measurements of nutritional markers.

During follow-up, detailed questionnaires on infant feeding, introduction of solid foods will be sent every three months to a sub-sample of the cohort while a summary of information about feeding will be obtained for the rest of the cohort at the six-week and one year interviews. Additional follow-up assessments of child's food intake and eating behavior will rely on questionnaires administered to the mother.

#### 2. Outcomes

##### Health outcomes

First, questionnaires and data from medical files will focus on the health of newborns at the maternity hospital, childhood disorders and mother's health, notably the course of pregnancy, perinatal period, health status of women at birth and postnatal depression. Self-administered questionnaires at the maternity unit will provide data on dietary habits and exposure to environmental pollutants during pregnancy.

Subsequent follow-up will focus on height and weight growth, sexual development, respiratory and allergy symptoms, occurrence of diseases and vaccinations, A medical questionnaire and examination of the child by a medical doctor at age 6 will provide anthropometric data (weight, height), information on visual and hearing acuity, functional respiratory capacity.

##### Child's cognitive and motor development

Information will be collected by interviewing parents using published developmental tools. For example, general child's development will be explored using the Child Development Inventories (CDI) [34] at 1 year. At 2 years, the emphasis will be put on early language development using the MacArthur Communicative Inventories. At 3 years, a cognitive abilities tool will be passed by the child during the home interview. Different scales are considered eligible for the 3 years interview such as the Bracken or the British Ability Scales (BAS [35]). At 6-7 years, it is project to use the Wechsler Intelligence scale [36] during the medical examination in order to have a health professional evaluation of the child's development.

##### Social outcomes

Face-to-face and phone interviews will cover topics such as the changes in family structure (divorce, reconstituted family, stepfamily, death, etc), the different factors involved in the socialization of the individual: the roles played by parents, institutions (schools, etc.), social relationships outside school and inter-generational relations. They will focus on the children's lifestyles through their living conditions, school attendance and careers, parents' incomes and profession and the transmission of cultural practices, before moving on to study social inequalities. They will also describe the living conditions of underprivileged children from a dynamic viewpoint by understanding how parents enter or leave a state of poverty or are maintained in it.

## Discussion

The main weakness lies in the absence of data collection before birth and during pregnancy, especially since the prenatal period has been highlighted as a sensitive period for various exposures [37]. Another weakness for any life course cohort is the difficulty of maintaining representativeness throughout the follow-up [38,39], but employing special methods such as a joint parameter-dependent selection model to account for non-ignorable dropout will allow us to control for the attrition process while studying the impact of determinants of children's life events and evolution [40].

One of the main strengths of the study is the statistical power and the large size of the cohort which should allow us to test a wide variety of hypotheses. The creation of this large-scale cohort will provide a representative sample of the general population. Other study strengths also include the prospective collection of data from different sources, which can facilitate cross-validation of specific information, the use of objective measures (biological samples, biomedical data from physical examinations, etc.) and standardized tools which allow international comparisons.

Another strength of the study is that it involves multiple scientific fields, leading to a greater understanding of the factors involved in child growth and their interactions. For example, a wide range of environmental factors are thought to have an impact on child health, extending well beyond chemicals, and including nutrition, lifestyle, parental health, or living environment. In particular the pluridisciplinary aspect of the ELFE study will facilitate a more detailed analysis of socioeconomic factors and family lifestyle. These factors are particularly important in the psychomotor development of the child, and their investigation will help in studying the impact of neuro-toxic chemical substances on a child's neurological development. Only a large longitudinal study can begin to discriminate between the complex influences of early life, particularly where chronology is important for example, in unravelling the influences of pregnancy, the physical environment, early family life, and that of the school. Properly conducted and interpreted, the study will produce invaluable information, not only to the proposing scientists, but also to countless researchers who will be able to interrogate the resulting data with a wide variety of research questions reflecting a range of hypotheses. From an international perspective, the comparative analyses that would ensue would be of immense public health interest.

## Competing interests

The authors declare that they have no competing interests.

## Authors' contributions

HL and GS designed the study, HL is the director of the project. HS analyzed the data, SV drafted the manuscript. SV, CB, CP organized and participated in the field investigation and were involved the study design and contributed as supervisor and provided all scientific and technical supports with the Elfe team. All authors approved the final manuscript.

## Pre-publication history

The pre-publication history for this paper can be accessed here:


